# Enhanced Lipid Production of *Chlorella* sp. HS2 Using Serial Optimization and Heat Shock

**DOI:** 10.4014/jmb.1910.10033

**Published:** 2019-11-01

**Authors:** Hee Su Kim, Minsik Kim, Won-Kun Park, Yong Keun Chang

**Affiliations:** 1 Department of Chemical and Biomolecular Engineering, Korea Advanced Institute of Science and Technology (KAIST), Daejeon 344, Republic of Korea; 2 Department of Chemistry and Energy Engineering, Sangmyung University, Seoul 03016, Republic of Korea; 3 Advanced Biomass R&D Center, Korea Advanced Institute of Science & Technology (KAIST), Daejeon 4141, Republic of Korea

**Keywords:** *Chlorella* sp. HS2, fed-batch cultivation, elemental analysis, heat shock stress, lipid productivity

## Abstract

*Chlorella* sp. HS2, which previously showed excellent performance in phototrophic cultivation and has tolerance for wide ranges of salinity, pH, and temperature, was cultivated heterotrophically. However, this conventional medium has been newly optimized based on a composition analysis using elemental analysis and ICP-OES. In addition, in order to maintain a favorable dissolved oxygen level, stepwise elevation of revolutions per minute was adopted. These optimizations led to 40 and 13% increases in the biomass and lipid productivity, respectively (7.0 and 2.25 g l^-1^d^-1^ each). To increase the lipid content even further, 12 h heat shock at 50°C was applied and this enhanced the biomass and lipid productivity up to 4 and 17% respectively (7.3 and 2.64 g l^-1^d^-1^, each) relative to the optimized conditions above, and the values were 17 and 14% higher than ordinary lipid-accumulating N-limitation (6.2 and 2.31 g l^-1^d^-1^). On this basis, heat shock was successfully adopted in novel *Chlorella* sp. HS2 cultivation as a lipid inducer for the first time. Considering its fast and cost-effective characteristics, heat shock will enhance the overall microalgal biofuel production process.

## Introduction

Recent microalgal biomass production has been limited to photoautotrophic cultivation because it is the only method to use photosynthesis; however, the scale of biomass production has shown limitations and inadequate feasibility [1, 2]. In response, heterotrophic microalga cultivation using organic carbon sources in closed systems has been considered as an alternative [3]. Inevitably, heterotrophic cultivation has drawbacks as well, such as insufficient economic feasibility caused by the utilization of carbon sources. Nonetheless, this can be compensated by the utilization of reasonable carbon sources and the production of value-added products [4, 5].

At the same time, most microalgae strains are accustomed to growing in phototrophic rather than heterotrophic conditions. Therefore, careful strain selection, adaptation, and serial optimization in heterotrophic cultivation are essential for massive biomass production. The ideal strain for heterotrophic cultivation should use various carbon and nutrient sources in order to utilize various low cost carbon sources or wastes/wastewaters [4, 6]. In addition, the potential of such strains to produce both biomass and lipids or other value-added products in heterotrophic cultivation is also important [7]. Therefore, better understanding of the traits that allow greater accumulation of a target product in specific conditions is necessary to design a culture system to maximize product yields [8].

While adaptation is not always necessary, it should be conducted for stable cultivation. Algae strains that normally grow in natural phototrophic cultivation seldom use glucose or other carbon sources. Although they sometimes have genes coding the enzymes that are part of a metabolism using organic carbon sources, the genes do not work effectively and the cells consequently show lower performance than they would have normally [3]. Therefore, in order to fully understand their ability, cell growth and lipid accumulation performance should be confirmed and cells needs to undergo adaptation and be optimized in the targeted system. Optimized conditions in the specific system also could save costs by reducing the cultivation period and enhancing the product yield with higher conversion yield [9, 10]. In this aspect, optimization of the given system is important not only for production but for feasibility.

The optimization starts from setting a system for the target strain or product. The system is decided based on data of final productivity and a techno-economic assessment. In the case of a certain strain, it will show inhibited growth when cells are exposed to a high concentration of nutrients, and therefore a fed-batch cultivation system would be a better option [11]. Fed-batch operation leads to high lipid density cultivation without this inhibition [12, 13]. In the fed-batch system, it is also necessary to consider better composition and combustion of the initial nutrients or feeding stock to achieve the highest productivity of biomass and product [11]. In addition, better uptake of nutrients and higher metabolic activity lead to an increase in oxygen demand in the fermenter [14]. In this case, the level of dissolved oxygen could be controlled by the oxygen transfer rate by increasing the mixing rate or increasing the concentration of supplied oxygen [15]. All these optimizations should be conducted step by step based on the cell growth performance or target product profile.

The recently isolated *Chlorella* sp. HS2 is a microalga having beneficial traits as a target strain for heterotrophic biodiesel production. It has been reported that *Chlorella* sp. HS2 can utilize various carbon sources and has higher cell density in both phototrophic (4.34 g l^-1^) and heterotrophic (7.71 g l^-1^) conditions [16]. It has tolerance to a wide spectrum of salinity (0 to 5%) and pH (3 to 10.5) [16]. It thrives in a wide temperature range (14 to 46°C), and can even tolerate temperatures of up to 60°C for a short time (~12 h). These tolerances to salinity, pH, and temperature are highly pursued characteristics in industries because microalgae sometimes must be subjected to stressful conditions to induce them to accumulate the target product [17-19]. The target production is not always enhanced with the biomass productivity. In the case of lipids, they start to accumulate when cell growth is stopped by stressful conditions [20]. If microalgae have tolerance to certain stressful conditions, then it is easier to increase the target product content by applying moderately stressful conditions. Additionally, these stressful conditions could be exploited for stepwise stress induction after an intense biomass production step.

In this study, *Chlorella* sp. HS2 was selected as an ideal heterotrophic microalga and optimized in a fed-batch cultivating system. For the initial optimization of biomass productivity, the fed-batch fermentation was compared with batch fermentation and enhanced by modification of the concentration of the nutrients, which was calculated by elemental analysis and ICP-OES data of the cell biomass composition. Second, stepwise elevation of RPMs was adopted to satisfy increasing oxygen demand as cells grew. Lastly, heat shock was applied as a more cost-effective and stepwise lipid inducer compared with conventional N limitation.

## Materials and Methods

### Strain and Maintenance Condition

*Chlorella* sp. HS2, which is a green microalga, was generously provided by Dr. Hee-Sik Kim at the Korea Research Institute of Bioscience and Biotechnology (KRIBB). The culture was maintained in a modified BG-11 medium that was previously optimized in a batch condition: 72 g glucose, 8 g NaNO_3_, 0.22 g K_2_HPO_4_∙2H_2_O, 1 g MgSO_4_∙7H_2_O, 0.02 g Na_2_CO_3_, 0.006 g ferric ammonium citrate, and 1 ml of the A5 solution per 1 L DI water [16]. The A5 solution consisted of 2.86 g H_3_BO_3_, 1.81 g MnCl_2_∙4H_2_O, 0.22 g ZnSO_4_∙7H_2_O, 0.39 g NaMoO_4_∙2H_2_O, 0.079 g CuSO_4_∙5H_2_O, and 0.05 g CoCl_2_∙6H_2_O per 1 L DI water. The maintaining conditions were 37°C and 120 rpm in a heterotrophic incubator.

### Heterotrophic Cultivation Conditions in the Fermenter

The seed culture was prepared in a 500 ml flask with 300 ml of the modified BG-11 described above. Cultivation was conducted in a shaking incubator at 37 °C and 120 rpm in the dark. The initial pH was adjusted to 7.0 using 1 N HCl. Fermenter scale cultivation was conducted using a 5-L jar fermenter (CNS Co. Ltd., Korea) containing 3 L of the modified BG-11. For the fed-batch cultivation, stock solutions of the modified BG-11 were used to maintain the concentration of glucose at around 20-40 g l^-1^. Culture conditions were 1 vvm air flow rate, 37°C, pH 7, and 300 rpm respectively. The initial concentration of the inoculum was 7.2 × 10^6^ cells ml^-1^, and the total cultivation period was ten days. Batch cultivation was also conducted with the same conditions except for the feeding.

For the optimized fed-batch cultivation, optimized BG-11 medium and stepwise increase in rpm were applied. The optimized BG-11 medium was calculated using a stoichiometry equation (Eq. 1) based on the cell composition data, where the cell condition was an exponential phase, from Elemental Analysis (EA).

(1) $$C_{6}H_{12}O_{6}+{2.085O}_{2}+{0.252\mathrm{NO}}_{3}^{-}\to {3.6\mathrm{CH}}_{1.88}O_{0.54}N_{0.07}+{2.616H}_{2}O+{2.4\mathrm{CO}}_{2}$$

The optimized BG-11 medium contained 15 g NaNO_3_ and 2.2 g K_2_HPO_4_∙2H_2_O per 1 L DI water and the other components were the same as in the modified BG-11 medium. Feeding was conducted using only glucose stock to maintain the concentration of glucose at around 20 to 40 g l^-1^. In the case of rpm, in order to minimize the negative effect from shear stress [21], stepwise rpm elevation was applied (started from 300 and increasing 100 rpm every 12 h until 600 rpm). Conditions of 1 vvm air flow rate, 37°C, and pH 7 were applied. Initial cell density was 7.2 × 10^6^ cells ml^-1^ and the total cultivation periods were ten days. All the cultivations were conducted twice.

### N Limitation and Heat Shock

In order to induce nitrogen limitation in the fermenter, 3 L of optimized BG-11 medium containing 7.5 g l^-1^ NaNO_3_ was used in a 5-L fermenter. Glucose feeding using only glucose stock was conducted to keep the concentration of glucose at around 20 to 40 g l^-1^. The temperature was kept at 37°C. In the case of heat shock, 3 L of optimized BG-11 medium containing normal 15 g l^-1^ sodium nitrate was used in a 5-L fermenter and only glucose feeding was adopted to maintain the concentration of glucose. The temperature was started at 37°C and increased to 50°C on day 7. For both nitrogen limitation and heat shock, culture was started with the same initial cell density (7.2 × 10^6^ cells ml^-1^), air flow rate, and pH, and stepwise rpm elevation was applied (1 vvm air flow rate, pH 7, starting from 300 and increasing 100 rpm every 12 h until 600 rpm). All the cultivations were conducted twice.

### Biomass and Total Lipid Analysis

For the dry cell weight (DCW), a 1-ml sample was centrifuged at 13,000 rpm for 3 min. After washing twice, cells were dried in a 90°C oven for 24 h. To measure the total lipid amount, 20 ml of the biomass samples was centrifuged at 4,000 rpm for 10 min. The pellets were washed twice with distilled water and then stored at −70°C in a deep freezer for more than 24 h. Frozen cells were lyophilized in a freeze dryer for up to three days. Total lipid was determined using the modified Folch method [8, 22]. The dried cell was grounded into powder by a bead beater. Chloroform/ methanol (2:1, v/v) (10 ml) was added into dried biomass (30 mg) and the mixture was sonicated for up to 4 h. After adding 2.5 ml of DI water, the sample was vortexed and centrifuged at 4,000 rpm for 10 min. The lower solvent phase was transferred to another glass vial through an organic solvent filter (PTFE membrane filters 0.2 μm, Germany) using a syringe. Subsequently, 4 ml of the filtered solvent was transferred to a pre-weighed aluminum dish and evaporated under a fume hood. After evaporation, the aluminum dish and total lipid were weighed. The lipid content was calculated using Eq. 2.


(2) $$\mathrm{Total}\mathrm{lipid}g\mathrm{of}\mathrm{oil}100g^{-1}\mathrm{sample})=\frac{(W_{L}-W_{D})\times V_{C}}{V_{P}\times W_{S}}\times 100$$

WD: weight of the aluminum dish, WL: weight of aluminum dish with lipid, WS: weight of the sample, VC: volume of chloroform, VP: volume of chloroform in the aluminum dish

### Glucose and Nitrate Analysis

The supernatant from the culture was filtered using a 0.2 µm syringe filter (Sartorius Stedim Biotech, Germany). A high-pressure liquid chromatograph (HPLC, Waters, USA) equipped with a refractive index detector (RI) was used to analyze the glucose concentration. The Aminex HPX-87H column (300 mm × 7.8 mm; Bio-Rad, USA) was maintained at 65°C with an eluent that contained 0.01 N, H2SO_4_.

The nitrate concentration was measured with an ion chromatograph (883 Basic IC Plus, Switzerland) with an anion column (Metrosep A Supp 5). The eluent, which consisted of 3.2 mM Na_2_CO_3_ and 1 mM NaHCO_3_, was supplied at a flow rate of 0.7 ml min-1 into the column for the analysis [23].

### Elemental Analysis and ICP-OES

For the elemental analysis and ICP-OES, cells at the exponential phase were used. The contents of C, H, N, S, and O were analyzed using an elemental analyzer (FLASH 2000 series, Thermo Scientific, USA) in the KAIST Analysis Center for Research Advancement [24]. Various metal ions were analyzed by ICP-OES (SPECTRO BLUE, Germany) in the Advanced Biomass R&D Center (ABC).

## Results and Discussion

### Comparison between Fed-Batch and Batch Cultivation Using Modified BG-11

**Fig. 1:**
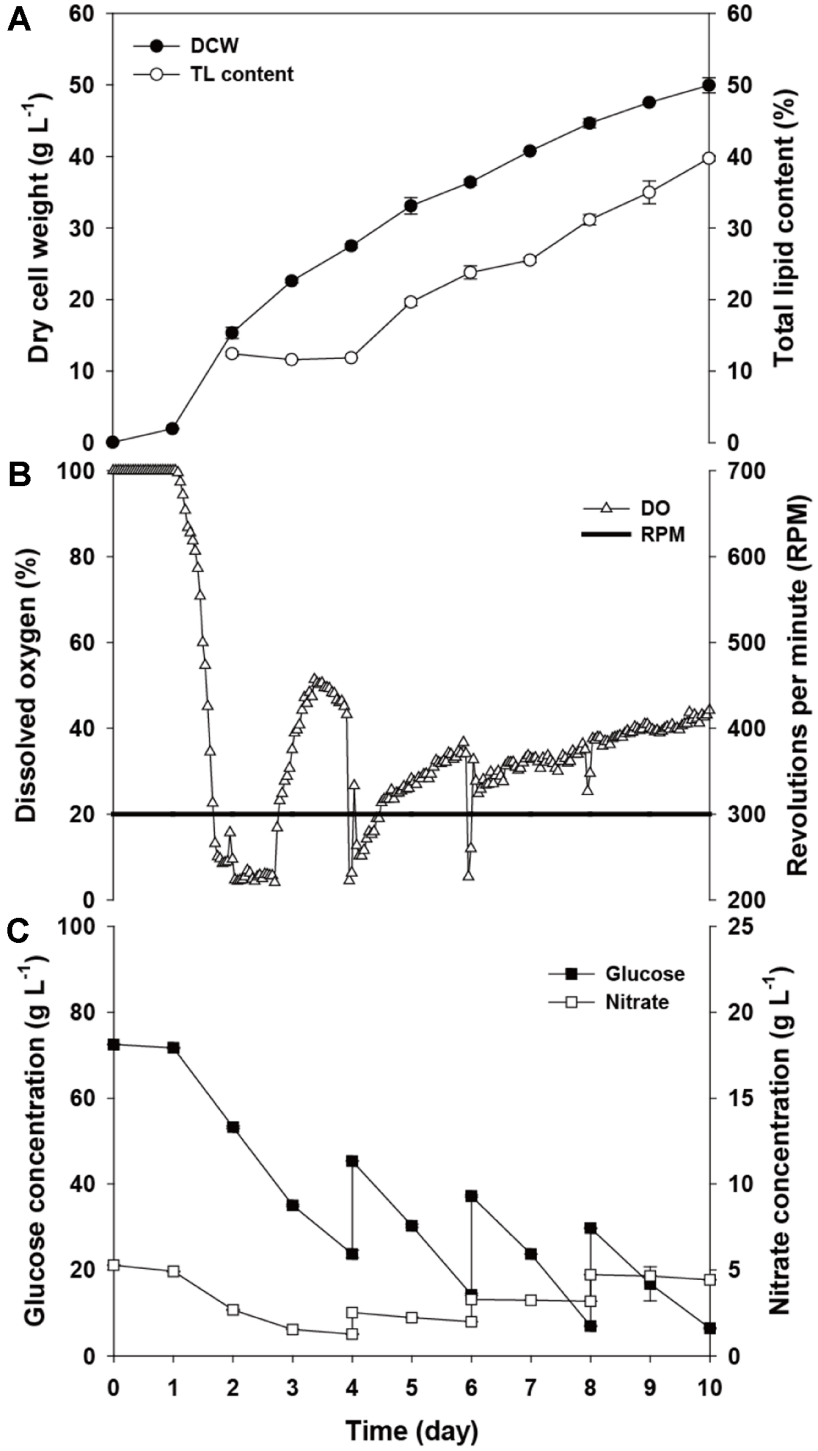
Profile of first fed-batch cultivation: (**A**) DCW and total lipid content; (**B**) DO and rpm; (**C**) glucose and nitrate concentrations.

The fed-batch process has been used as the general method of biomass and pharmaceutical product production in the industry because of the characteristics of easy scale-up and stable production [12, 13, 25]. In this study, lipid production from a heterotrophic microalga was tested intensively from the optimization of culture conditions to the second step, lipid induction. First, a comparison between conventional batch cultivation and fed-batch cultivation was performed. Modified BG-11, which was optimized in previous research, was used as the control medium [16]. For the feeding condition, the same modified BG-11 stock was used to check the limitations of the current conditions of the fermenter. If all conditions were sufficient, then there would be no improvement in fed-batch over batch cultivation. As shown in Fig. 1A, fed-batch cultivation of *Chlorella* sp. HS2 showed superior dry cell weight (50.0 ± 0.7 g l^-1^) and lipid content (39.8%) compared to batch cultivation (35.3 g l^-1^ biomass and 26.3% lipid content, Fig.S1A). Although fed-batch cultivation consumed a greater amount of glucose and nutrients, considering the better biomass and lipid productivity (41.6% increase in biomass productivity and 114% increase in lipid productivity), economic feasibility could be enhanced by short operation time, using organic waste and wastewater, and having more efficient harvest yield [26-28]. Therefore, fed-batch could be a better production method for massive production of biodiesel.

However, there are two factors to consider: an insufficient level of dissolved oxygen and incomplete consumption of nitrate. A lower level of dissolved oxygen is common in heterotrophic cultivation owing to the respiration caused by active cell metabolism. However, the level of dissolved oxygen should be controlled at a certain level because it can determine the glucose consumption and will decide the maximum achievable biomass yield.

Generally, dissolved oxygen (DO) is simply the measured concentration of dissolved oxygen in the media, and thus ΔDO is the most appropriate parameter to represent the oxygen utilization by the cells. For the initial two days, ΔDO showed negative values, which indicates that oxygen utilization exceeded the supply (Fig. 1B). For the next two days, both the DO as well as ΔDO remained close to 0, which means that the culture was depleted of oxygen and the growth could be bottlenecked by the oxygen supply. As such, the growth rate of the culture gradually decreased from day 2 (7.5 to 5.0 g l^-1^ day^-1^). Although decreased DO inhibited cell growth with an insufficient oxygen supply, interestingly, DO showed a slow increase from day 2 with sudden decreases at day 4, 6, and 8. The increase of DO continued until the end of the cultivation after the cells reached the optimum level of growth. It can be assumed that overall growth lost its activity due to the increase in the aged cell and insufficient oxygen supply. Whereas DO continued to increase and fluctuate after day 2, a certain range of DO is necessary for stable cell growth [29, 30]. Therefore, an adequate oxygen supply method should be applied to overcome the lack of oxygen.

In addition, as shown in Fig. 1C, nitrate in the medium accumulated during feeding of BG-11 stock. Fed-batch cultivation was conducted to enhance cell growth by supplying sufficient nutrients. However, the amount of supplied nutrients should be proportional to the consumption to prevent the negative effect of a high concentration of nutrients on cell growth and the lipid accumulation mechanism [25]. Thus, in order to provide cells with the best conditions for lipid production, optimization of the level of other nutrients, as well as nitrate for a fed-batch condition, is necessary.

### Deciding the Amount of Nutrient Supplementation Based on Biomass Analysis and Aeration Method

In order to optimize the amount of supplied nutrients including nitrate, elemental analysis and Inductively Coupled Plasma Optical Emission Spectrometry (ICP-OES) were conducted on *Chlorella* sp. HS2 biomass. Cell biomass was prepared in favorable conditions to determine the cell composition that shows the most robust growth. Cell biomass-based prediction could be a better method than prediction based on the consumption profile in the medium, because the concentration of the nutrients in the medium may not be accurate due to secreted chemicals from the cells and various interferences [31, 32]. Therefore, elemental analysis was used to check the major requirements: carbon and nitrogen. ICP-OES was meanwhile used to measure phosphorus and the other minor and trace elements. The elemental analysis of biomass of *Chlorella* sp. HS2 reveals the exact weight-based ratio of C:N:H:S:O (51.19:4.17:7.67:0.35:36.63), as presented in Table 1. Based on the results, the empirical formula of *Chlorella* sp. HS2 biomass was calculated as CH_1.88_O_0.54_N_0.07_. Assuming that glucose, sodium nitrate, and oxygen were provided to make the biomass of *Chlorella* sp. HS2, the biomass can then be represented by the following mass balance equation as Eq.1.

First, the required glucose for 60 g l^-1^ biomass production was calculated as 76 g l^-1^ through Eq. 1. In the case of glucose, it could be used as an energy source as well as a carbon source, and therefore cells require a greater amount of glucose than the calculated amount based on Eq. 1. However, the supplementation of too much glucose could have a negative effect on cell growth, such as osmotic shock [25], and thus it was decided to start with 76 g l^-1^ of glucose and add additional glucose to keep the glucose concentration at around 20 to 40 g l^-1^, which is the ideal range for cell growth [33, 34].

In addition, the amount of sodium nitrate required for the production of biomass of 60 g l^-1^ was also calculated as 15 g l^-1^ based on Eq. 1. While this number was substantially larger than in the previous fed-batch test, optimization of nutrients and modification of the aeration method would enhance the consumption rate, and therefore the nitrate consumption profile was tested later, as described in the following section. The third major nutrient, the phosphorus source, was examined by using ICP-OES. The amount of dipotassium phosphate needed for 60 g l^-1^ of biomass was calculated to be 2.2 g l^-1^ given that the phosphorus composition in the cell biomass was about 6.7 mg/g biomass (Table 1). For dipotassium phosphate, it is noteworthy that microalgae tend to assimilate a greater amount of phosphate than the amount they actually metabolize [35-37]. In the case of an excessive amount of phosphate, cells store it in the form of phosphate granules, and this makes it difficult to confirm the exact amount of phosphate they need by prediction using elemental analysis. Therefore, the amount of phosphate calculated based on the biomass composition is sufficient for the whole cultivation period. The addition of 2.2 g l^-1^ of phosphate was thus selected. Minor nutrients and trace elements were calculated by using the same method as employed for phosphorus, and it was shown that their concentrations in the modified BG-11 were sufficient. Therefore, the initial conditions were decided as the addition of 72 g l^-1^ glucose (supported with feeding, too), 15 g l^-1^ sodium nitrate, and 2.2 g l^-1^ phosphate while the other components were the same as in the modified BG-11 medium.

In order to increase the DO level in the fermenter, numerous strategies such as supplying only O_2_ and modifying the baffle or bubble size have been suggested [15]. Among the various methods, the most effective method for application in our fermenter is rpm manipulation [38]. However, the shear stress per cell will be greatly increased when microalgae are cultivated at high rpm in the lag phase [21, 39] and this causes a severe negative effect on the cell growth [30]. In addition, at low cell density, the oxygen demand is also low, and therefore a real time increase in the rpm that is appropriate to each cell density level is required [29, 40]. Thus, we chose to use stepwise rpm elevation for the fed-batch fermenter.

### Upgraded Fed-Batch Using Optimized Nutrient Supplementation and Stepwise Elevation of RPM

**Fig. 2:**
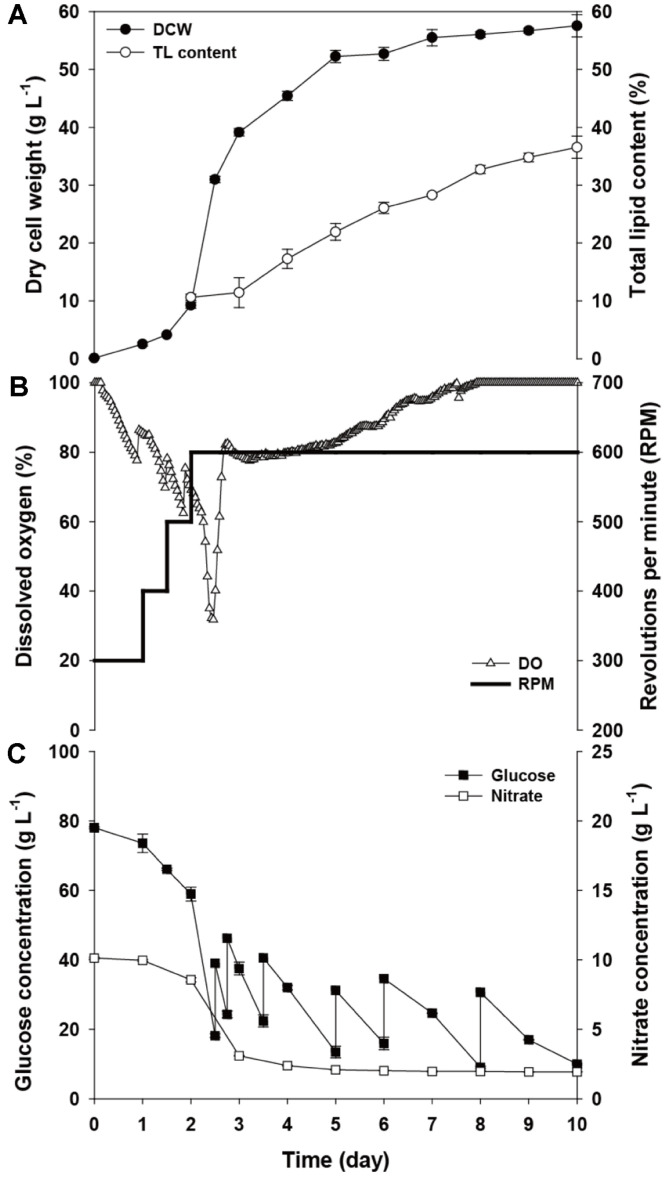
Profile of the fed-batch cultivation using optimized nutrients and stepwise rpm elevation: (**A**) DCW and total lipid content; (**B**) DO and rpm; (**C**) glucose and nitrate concentrations.

The first fed-batch was successfully carried out and showed higher biomass and lipid yield than batch cultivation. However, problems of reduced DO level and accumulation of nitrate were confirmed, and therefore we applied optimized nutrient supplementation and stepwise rpm elevation to the fed-batch cultivation of *Chlorella* sp. HS2, as presented in Fig. 2. *Chlorella* sp. HS2 in the optimized conditions showed a maximum DCW of 57.6 ± 1.3 g l^-1^ and total lipid content of 36.6% at day 10 (Fig. 2A). The maximum biomass increased slightly from 50.0 (Fig. 1A) to 57.6 g l^-1^ (Fig. 2A) and, considering that biomass reached the stationary stage at day 5, the biomass productivity increased by about 40% from the results of the first fed-batch process. Also, the lipid productivity increased from 1.99 to 2.11 g l^-1^d^-1^.

The enhanced performance was mainly the result of the application of stepwise rpm elevation, as shown in Fig. 2B. It was successfully adopted before the depletion of DO and, as intended, it not only prevented the DO value from decreasing below 20% but also kept it at around 80% when cells actively used the glucose at day 2 to 5. Compared to the fed-batch cultivation with fixed rpm (Fig. 1B), the DO level was well maintained and this brought enhanced consumption of glucose and nitrate (Fig. 1C). When the rpm increased, total glucose consumption greatly increased from 135.1 (initial conc. 72.4 and total fed conc. 62.7 g l^-1^, Fig. 1C) to 163.8 (initial conc. 78 and total fed conc. 85.8 g l^-1^, Fig. 2C) g l^-1^. This increase in glucose consumption was coupled with enhanced nitrate consumption from 4.9 (Fig.1C) to 8.1 (Fig.2C) g l^-1^. After day 5, nitrate consumption stopped; however, glucose consumption continued (40.9 g l^-1^). During this period, there was no significant cell growth, but the lipid content kept increasing until reaching 36.6%. In view of the biomass production, the biomass productivity was 10.3 g l^-1^d^-1^ at day 5, and therefore the cultivation period could be shortened. However, considering the lipid production with the aim of biodiesel production, the eight days of cultivation would be optimal for any subsequent process.

The application of optimized nutrient supplementation and stepwise rpm elevation successfully enhanced the yield and productivity of biomass and lipid of *Chlorella* sp. HS2. While biomass production was clearly enhanced, as the lipid content continued to increase until the end of the cultivation, there is still room to increase the lipid productivity. For better lipid productivity, an enhanced and fast lipid accumulation process is necessary. Thus, additional lipid induction processes were applied.

### Lipid Induction Test Using N Limitation and Heat Shock

Lipid induction after cell growth has been studied extensively because of the relationship between cell growth and lipid accumulation [20]. Thus far, many kinds of methods have been applied for lipid induction, but the response to these methods varied with each condition and strain [41]. In this study, we tested two methods: N limitation and heat shock.

**Fig. 3:**
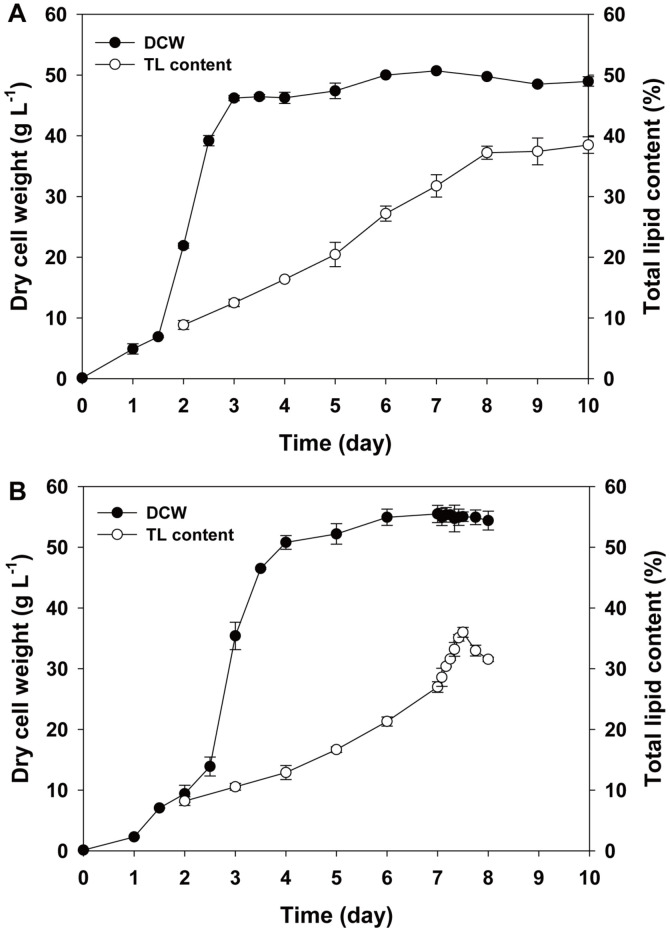
Comparison of cell growth and total lipid content between N-limitation and heat shock stress induction : (**A**) DCW and total lipid content under N-limit stress; (**B**) DCW and total lipid content under heat shock stress.

While N depletion is the most common lipid-inducing method [32, 42], it has only been applied on a lab-scale. The reason is the additional cost for cell harvesting and the use of an N-free medium. Therefore, N limitation, which gives a smaller amount of nitrogen source depleted in the middle of cultivation, could be a better option for large fermenter scale cultivation. For this, lower concentrations of sodium nitrate (12 and 7.5 g l^-1^) were tested under the same upgraded conditions noted above. As shown in Fig. 2C, when 15 g l^-1^ sodium nitrate was supplemented, 81% was consumed, and therefore 12 g l^-1^ of sodium nitrate was anticipated to be the optimum concentration for both biomass production and lipid accumulation. However, 12 g l^-1^ of sodium nitrate was not consumed completely during the whole cultivation period (Fig. S2). In response, 7.5 g l^-1^ of sodium nitrate was adopted and it showed depletion at day 3 (Fig. S2). As a result, *Chlorella* sp. HS2 in the N-limited condition using 7.5 g l^-1^ sodium nitrate produced a maximum DCW of 49.8 ± 0.1 g l^-1^ and total lipid content of 37.2% at day 8 (Fig. 3A). Although there was a decrease in the maximum biomass yield (14%), the lipid content increased by about 5% relative to the upgraded fed-batch (Fig. 2A). With this result, the lipid productivity also increased from 2.11 to 2.31 g l^-1^d^-1^, as intended.

The other method of increasing lipid content is heat shock. Modifying the temperature also has been used as an inducer of lipid accumulation [43]. Generally, temperature modification has entailed the application of cold stress because lower temperature only causes metabolic change or slowed metabolism, whereas higher temperature, especially beyond a certain range, often causes denaturation of the enzymes and results in cell death [43]. The special characteristic of *Chlorella* sp. HS2 is its thermal tolerance to higher temperature up to 60°C. It is noteworthy that the modification of temperature, which could have a negative effect on biomass productivity, is conducted as an additional induction process after the end of biomass production. Therefore, it can easily be added after the previous process to obtain the optimum conditions for biomass productivity and is easy to apply, unlike the N limitation method. Heat shock was conducted with temperature change from 37 to 50°C at day 7 and the other conditions were the same as employed for the upgraded fed-batch cultivation. As shown in Fig. 3B, cell growth and the lipid accumulation profile before the heat shock were almost the same as in the case of the upgraded fed-batch (Fig. 2A). During the heat shock, we checked the dry cell weight and lipid content every 2 h, and there were no significant changes in dry cell weight. However, the lipid content kept increasing from 27 to 36% until 12 h and thereafter decreased to 31.5%. Therefore, the highest lipid content and productivity was achieved at day 7.5 (36% and 2.82 g l^-1^d^-1^, respectively).

Two lipid-accumulating processes are compared and heat shock provided better performance in terms of both biomass and lipid yield with shorter induction time. For the N limitation, there was limited biomass production caused by lower nitrate concentration, and for the lipid accumulation, the cells need more time to consume intercellular nitrate and turn on the lipid-accumulating metabolism. However, heat shock treatment, which could be applied with exact timing, showed the highest biomass productivity and the treatment takes only 12 h to induce lipid accumulation. Therefore, heat shock could be well utilized in this system. Thus, it is noteworthy that the powerful and fast lipid induction performance of heat shock showed strong potential for biofuel production from *Chlorella* sp. HS2.

As a result, we applied optimization and additional lipid accumulation methods, as delineated in Table 2. As the optimization and application processes progressed, biomass productivity and lipid content were enhanced. First, switching to fed-batch showed enhanced biomass productivity (3.5 to 5.0 g l^-1^d^-1^) and total lipid content (26.3 to 39.8%), finally resulting in doubled total lipid productivity (0.93 to 1.99 g l^-1^d^-1^). In addition, improved nutrient supplementation and stepwise elevation of rpm were successfully adopted and increased biomass productivity (5.0 to 7.0 g l^-1^d^-1^). There was little decrease in the total lipid content (39.8 to 32.2%), but the increase in biomass productivity compensated for this and showed higher total lipid productivity (1.99 to 2.25 g l^-1^d^-1^). Finally, application of heat shock for lipid accumulation enhanced the biomass productivity (7.0 to 7.3 g l^-1^d^-1^) and increased the total lipid content (32.2 to 36%), and this resulted in 2.64 g l^-1^d^-1^ total lipid productivity. These values are substantially higher compared to the other Chlorella spp. (Table 3). *Chlorella protothecoides* is one of the most widely examined microalgae in industrial-scale culture research [12, 13, 33], because they have very good lipid accumulation capability (55.2, 46.1, and 32.8%, respectively). However, their biomass yields were relatively lower (15.5, 15.5, and 47.1 g l^-1^, respectively) than the results obtained with *Chlorella* sp. HS2 in the present study. The biomass productivity of *Chlorella* sp. HS2 was slightly lower at 0.39 g l^-1^d^-1^, but accompanied fairly high levels of biomass accumulation and lipid content. Therefore, the lipid productivity of *Chlorella* sp. HS2 was high. Each lipid productivity (g l^-1^d^-1^) was 1.05, 0.93, and 2.06, based on the 3-L working volume. Thus, it is noteworthy that the performance of lipid productivity of *Chlorella* sp. HS2 was achieved by its beneficial traits for heterotrophic cultivation, adequate optimization, and newly applied heat shock, which have not been used before well.

In order to increase the total lipid productivity for biodiesel production from microalgae, serial optimization and stepwise lipid induction were applied in heterotrophic fed-batch cultivation. First, efforts were made to improve the nutrient utilization and oxygen transfer in the fermenter, which resulted in increased biomass productivity of *Chlorella* sp. HS2. Through an elemental analysis and ICP-OES on cell biomass, the theoretically required amounts of nutrients were calculated and stepwise rpm elevation was selected. These optimizations increased the total lipid productivity by enhancing the biomass productivity. This has been supported by a comparison between stepwise lipid-inducing methods, N limitation, and heat shock to provide more lipid accumulation. As a result, heat shock was successfully adopted in a lipid induction process in heterotrophic fed-batch of *Chlorella* sp. HS2 for the first time. Thus far, there have been fewer endeavors involving heterotrophic microalgae cultivation, because phototrophic cultivation is the mainstream method of algal cultivation. However, recent circumstances in the algal industry necessitate a more stable and feasible process to produce target products. In this situation, actual improvement of growth performance and application of heat shock, which is an easy and rapid inductor, will bring novel applications involving fermentation of microalgae through industrial-scale production.

## Supplemental Materials



Supplementary data for this paper are available on-line only at http://jmb.or.kr.

## Figures and Tables

**Table 1:** Elemental analysis of biomass of *Chlorella* sp. HS2 in this study.

	Heterotrophic cultivation
Elements	*Chlorella* sp. HS2
C (%, w/w)	51.19 ± 0.10
N (%, w/w)	4.17 ± 0.01
H (%, w/w)	7.67 ± 0.09
S (%, w/w)	0.35 ± 0.01
O (%, w/w)	36.63 ± 0.18
The empirical formula of algal biomass	CH_1.88_O_0.54_N_0.07_
Molecular weight (g)	23.50
P (mg/g)	6.7 ± 0.05
Mg (mg/g)	0.5 ± 0.06
Cu (mg/g)	0.001 ± 0.0003
Ca (mg/g)	0.24 ± 0.02
Fe (mg/g)	0.05 ± 0.007
Zn (mg/g)	0.0002 ± 0.00001
Mn (mg/g)	0.00008 ± 0.000001
Co (mg/g)	0.0009 ± 0.00007

**Table 2:** Summary of heterotrophic cultivation results in this study.

Operation	Cultivation period (day)	Biomass productivity (g l^-1^d^-1^)	Total lipid content (%)	Total lipid productivity (g l^-1^d^-1^)
Batch	10	3.5	26.3	0.93
Fed-batch	10	5.0	39.8	1.99
Upgraded fed-batch (nutrient and rpm)	8	7.0	32.2	2.25
Upgraded fed-batch + heat shock	7.5	7.3	36.0	2.64

**Table 3:** Comparison of various *Chlorella* spp. in the heterotrophic fed-batch cultivation.

Microalgae	*Chlorella protothecoides*	*Chlorella protothecoides*	*Chlorella protothecoides* UTEX 256	*Chlorella* sp. HS2
Substrate	Glucose	Glucose	Glucose	Glucose
Biomass (g l^-1^)	15.5	15.5	47.1	55.0
Lipid content (%)	55.2	46.1	32.8	36.0
Biomass yield (g DCW/ g substrate)	0.48	0.40	0.43	0.39
Biomass productivity (g l^-1^d^-1^)	1.40	2.02	6.28	7.33
Lipid productivity (g l^-1^d^-1^)	1.05	0.93	2.06	2.64
Reference	Xu *et al*., 2006	Li *et al*., 2007	Chen *et al*., 2012	In this study^*^

^*^Upgraded fed-batch and heat shock were operated.
