# Supplementary material for: Enhanced Lipid Production of Chlorella sp. HS2 Using Serial Optimization and Heat Shock
Source: J Microbiol Biotechnol. 2019 Nov 1;30(1):136–45. doi: 10.4014/jmb.1910.10033 (PMC9745661; doi:10.4014/jmb.1910.10033)

J. Microbiol. Biotechnol.
https://doi.org/10.4014/jmb.1910.10033

Figure S1. Profile of batch cultivation: (A) DCW and total lipid content; (B) glucose and nitrate concentrations.

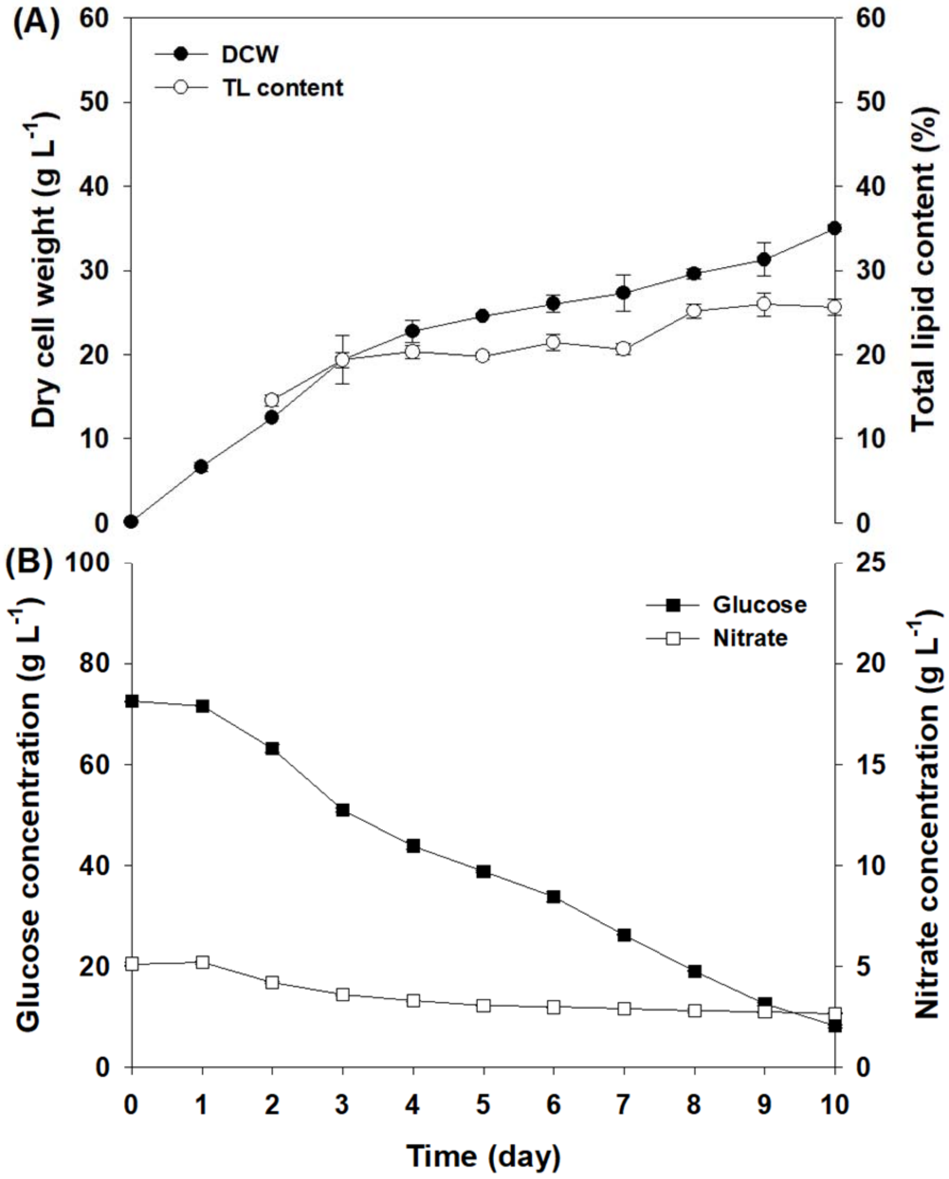

Figure S2. Nitrate profile of fed-batch cultivation with 12 and 7.5 g L^{-1} nitrate using optimized nutrients and stepwise RPM elevation

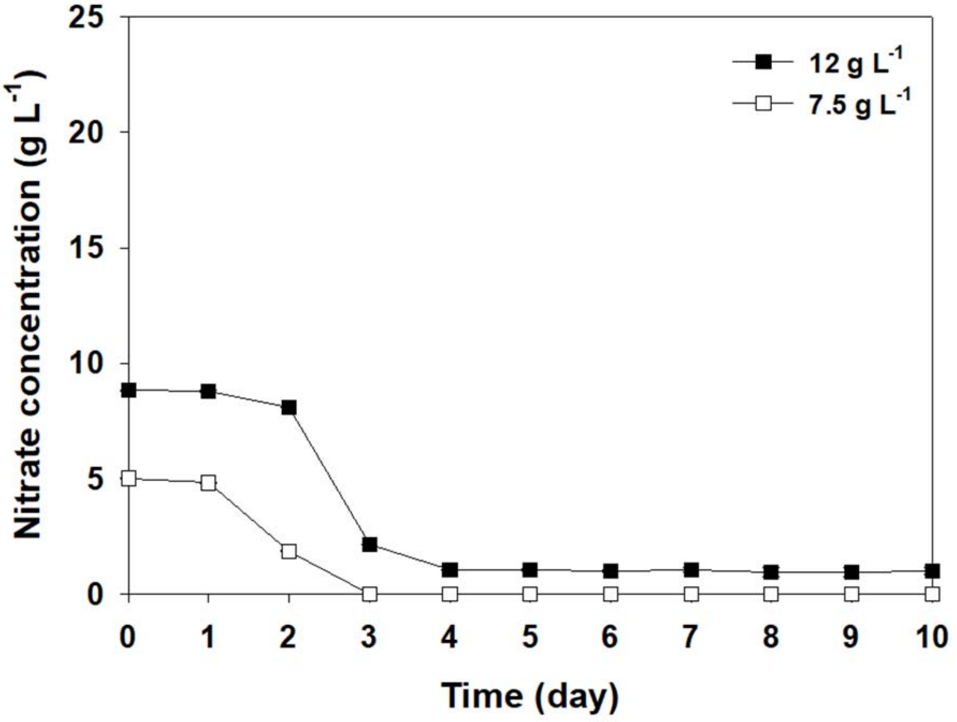

Supplement: Supplementary file 1 [file JMB-30-1-136-supple.pdf]
